# Differential effect of mild and severe pulmonary embolism on the rat lung transcriptome

**DOI:** 10.1186/s12931-016-0405-9

**Published:** 2016-07-19

**Authors:** John Zagorski, Jeffrey A. Kline

**Affiliations:** Department of Math and Sciences, Gaston College, Dallas, NC 28014 USA; Department of Emergency Medicine and Department of Cellular and Integrative Physiology, Indiana University Medical School, 720 Eskanazi Avenue, Indianapolis, IN 46202 USA

**Keywords:** Lung, Pulmonary hypertension, Inflammation, Microarray, GeneSifter, DAVID, Fibrinolysis, Thrombolysis

## Abstract

**Background:**

Pulmonary thromboembolism (PTE) is a common diagnosis and a leading cause of cardiovascular morbidity and mortality. A growing literature has associated PE with systemic inflammation, and global hyper-coagulability, which contribute to lung remodeling and clot recurrence. The source and mechanism of inflammation remains unstudied. In humans, inhibition of cholesterol synthesis with statins decreases biomarkers of inflammation. We test the differential effect of pulmonary vascular occlusion during mild and severe pulmonary embolism on the lung transcriptome.

**Methods:**

Experimental PE was induced in adult male rats by injection of 25 micron polystyrene microspheres into the jugular vein. The effect of Mild PE, (2-h right ventricular systolic pressure [RVSP] normal, 18-h RVSP 44 mmHg) and Severe PE (2-h RVSP > 50 mmHg; 18-h RVSP 44 mmHg) on lungs was assessed by measuring transcriptome-wide changes in gene expression by DNA microarrays.

**Results:**

Severe PE was associated with a large change in lung gene expression and in the expression of KEGG pathways and other gene functional annotation groups. Mild PE was also associated with a large number of significant changes in gene expression and in the expression of KEGG pathways and gene functional annotation groups, even after only 2 h of PE. Up-regulated pathways included increased adipocytokine, chemokine and cytokine signaling as well as cholesterol synthesis.

**Conclusions:**

Mild PE without acute pulmonary hypertension (PH) increased lung gene expression of inflammatory pathways, including increased cholesterol synthesis. These data indicate that even mild persistent pulmonary vascular occlusion is capable of inciting an inflammatory response from the lung. These data imply the detrimental effect of unresolved pulmonary obstruction from PE.

**Electronic supplementary material:**

The online version of this article (doi:10.1186/s12931-016-0405-9) contains supplementary material, which is available to authorized users.

## Background

Pulmonary embolism (PE) is a common and potentially lethal disease occurring in about 600,000 patients each year in the U.S., leading to as many as 60,000 deaths [1–5].

The most common form of PE is pulmonary thromboembolism (PTE), which results when blood clots, often formed in the deep vasculature of the legs, detach and enter the venous circulation. Circulating clots pass through the right heart and enter the lungs via the pulmonary artery, eventually lodging within the pulmonary vascular tree causing varying degree of pulmonary vascular occlusion and increased pulmonary vascular resistance. PE results in both acute and chronic sequelae. Acute PE causes a sudden increase in RVSP and acute PH, leading to right ventricular (RV) damage and dysfunction. A small subset of patients with unresolved PE go on to develop pulmonary vascular remodeling and RV hypertrophy which develops over time to produce chronic thromboembolic pulmonary hypertension (CTEPH) [6]. A hallmark of CTEPH includes persistent pulmonary vascular occlusion, and a widespread inflammatory response [7–11]. Increased lung inflammation has been implicated as a mechanism of reduced angiogenesis, and for increased hyper-coagulability, leading to recurrent PE [10, 12, 13]. Recurrent PE is a major risk factor for CTEPH development [8, 14]. Potential causes of inflammation include cells and molecules liberated by clots themselves [15], direct interaction of fibrin and the vessel wall [16], and the effect of shear on the vessel wall and platelets resulting in microparticle formation [9, 17]. Pulmonary vascular occlusion, with deprivation of blood flow to the lung also triggers a brisk inflammatory response [18].

We have previously described a rat model of PE induced by injection of 25 micron polystyrene microspheres into the right jugular vein [18–25]. Although microsphere PE lacks many of the characteristics of PTE such as platelet activation and thrombosis, it does faithfully produce the desired features of pulmonary ischemia and, at high doses, pulmonary hypertension. Rat lungs receiving doses of microspheres that produced acute PH (Severe PE, right ventricular systolic pressure [RVSP] ≈50 mmHg and 10 % animal mortality) had a >5-fold increase in recoverable bronchoalveolar lavage (BAL) neutrophils compared to control rats, indicating a neutrophilic inflammation [18]. Strong neutrophil chemotactic activity was measured in isolated alveolar lavage fluid and this activity was inhibited greater >50 % by treatment with anti-rat CXCL1 antibody. Rat lungs embolized with a lower dose of microspheres (Mild PE, normal RVSP 2-h post-PE and zero mortality), did not show neutrophil or protein accumulation in alveoli, but did show elevated expression of the chemokine genes CXCL1, CXCL2, CXCL3 and CCL2 [18]. These latter data led to a realization that Mild PE was sufficient to induce a pro-inflammatory environment within lung tissues, at least at the level of gene expression, and that lungs might be more sensitive to Mild PE than hearts. In this present study, the transcriptome-wide effect of Mild PE and Severe PE on rat lungs has been examined using DNA microarrays. The primary question was to answer the question whether whether Mild PE, which has been observed to be benign to RV dysfunction and inflammation [20–23], had a disproportionately worse effect on lungs.

## Methods

### Animal care

Experiments were done on male Sprague–Dawley rats weighing between 375 and 400 g. All experiments were conducted in accordance with the NIH Guide For the Care and Use of Laboratory Animals and were approved by the Institutional Animal Care and Use Committee (IACUC) of Carolinas Medical Center, Charlotte NC (4-02-01A and 11-00-01A). Prior to use, rats had ad libitum access to food and water.

### Pulmonary embolism model

PE was induced in rats by intra-jugular vein injection of 25-micron polystyrene microsphere beads (Duke Scientific #7525A, Palo Alto CA) as previously published [18–25]. Anesthetized rats were injected with either 1.3 or 2.0 million microspheres/100 g body weight to produce Mild PE and Severe PE, respectively. Control rats were injected with 0.15 ml/100 g of 0.01 % Tween 20 (the resuspension vehicle for microspheres), which was equivalent in volume to the Severe PE dose of microspheres. These treatment groups are referred to as “Vehicle” or “Veh”.

### Microarray analyses

Lung tissue samples from whole right lung lobes used for this study are the same as those collected for a previously published study [18]. In that study, Mild PE produced 2-h RVSP that was not statistically significant from RVSP in control rats (mean 39 mmHg for Mild PE verses mean 32 mmHg for controls, *p* > 0.05) while 2-h Severe PE caused elevation of RVSP to >50 mmHg [18]. Lungs were also collected from rats after 18-h of PE but RVSP was not measured. In subsequent studies, Mild PE was shown to cause a rise in 18-h RVSP to mean 44+/−1.3 mmHg (*p* < 0.05 relative to vehicle) while the RVSP in 18-h Severe PE was shown to fall from a peak of >50 mmHg to 44+/−0.9 mmHg [20, 22]. We have consistently concluded that Severe PE is associated with PH for the full time course of 2–18 h while Mild PE consistently shows PH only at the 18-h time. RNA was prepared from crushed whole right lung tissue which had been stored at −80 °C using the acid-phenol guanidinium isothiocyanate method of Chomczynski and Sacchi [26] followed by a second round of purification on RNeasy columns (Qiagen, Germantown, MD). Total lung RNA was prepared for microarray hybridization by standard Affymetrix procedures as previously described and checked for RNA integrity on agarose gels prior to use [23, 24]. Fragmented cRNAs were then hybridized to Affymetrix Rat Genome 230 v2.0 microarrays, washed and fluorescently stained in the Affymetrix Fluidics Station 400 using Affymetrix procedures. Each array was scanned twice by an Agilent Gene Array Scanner G2500A (Agilent Technologies, Palo Alto, CA).

Microarray data were initially analyzed with GeneSifter web-based software (Geospiza, Seattle, WA; (http://www.genesifter.net). Affymetrix “.cel” files were up-loaded to the GeneSifter web site using GC-RMA normalization into “Pair-wise” and “Project” folders for access to *t*-test and ANOVA statistical methods, respectively. A 2-way ANOVA was used to initially compare the six treatment groups using time as the first factor (2-h and 18-h) and microsphere dose as the second factor (Vehicle, Mild PE, Severe PE). A 1.5-fold expression difference threshold and Benjamini and Hochberg correction for false discovery (*p* < 0.05) was used as “pass” criteria. Genes that passed any one of the criteria of time, dose or interaction were accepted. The 6 sample groups used in the 2-way ANOVA were then subjected to hierarchical clustering to determine the similarities of the groups using the GeneSifter “Cluster” function.

Separate 1-way ANOVAs were used to compare the three 2-h treatment groups and three 18-h treatment groups for genes with related expression patterns based on the factor of microsphere dose (1.5-fold expression difference threshold relative to 2-h vehicle and 18-h vehicle groups as controls, respectively, Benjamini and Hochberg correction for false discovery, *p* < 0.05). Clustering of genes within the 2-h and 18-h ANOVAs were done using the GeneSifter PAM function (Partitioning Around Medoids) with a user-defined 12-cluster output. PAM searches a gene list for groups of genes (clusters) within the list that have a characteristic that is shared by all genes within that group but different from genes within other groups (hence, “clustering”). The characteristic used by GeneSifter was the pattern of expression each gene showed for the Vehicle, Mild PE and Severe PE treatments. GeneSifter allowed the user to specify the number of clusters that the entire gene list would be sorted into. For the analyses in Figs. 2 and 3, the 2-h and 18-h gene lists were sorted into 12 clusters. This number of clusters was determined empirically as the fewest number of clusters which yielded Mild PE-selective and Severe PE-selective expression patterns for the 2-h ANOVA data. These patterns were defined as expression patterns in which the change in expression from control was maximal between Vehcile and Mild PE and between Mild PE and Severe PE, respectively.

Pairwise comparison of treatment groups was done with GeneSifter using 2-sided unpaired t-tests with a 1.5-fold expression difference threshold relative to vehicle groups and with Benjamini and Hochberg correction for false discovery, *p* < 0.05.

Excel spreadsheet exports of 2-h and 18-h GeneSifter t-tests were used to prepare gene lists for further analysis using DAVID (Database for Annotation, Visualization and Integrated Discovery; http://david.abcc.ncifcrf.gov; [27, 28]). Lists of official gene symbols were first separated into new lists of up-regulated and down-regulated probesets from the pairwise *t*-test spreadsheets in Additional file 2A-C (2-h data) and 2D-F (18-h data). The six possible combinations of treatment times and PE doses were examined: 2-h and 18-h Mild PE verses Vehicle, Severe PE verses Vehicle, and Mild PE verses Severe PE expression. “UP” and “DOWN” lists of official gene symbols were then separately pasted into the search input in DAVID. DAVID analyzes gene lists and identifies all of the annotations that are attributed to each gene in the list. Gene annotations are categories of gene function, protein structural features, biochemical pathways and other shared gene or protein properties that are manually given by database curators to all genes and proteins. DAVID then produces several types of output that group the members of the input gene list into these annotations and determines statistical significance by comparing the number of genes from the submitted gene list that are present in each annotation with the total number of genes that are in the annotation (this is an “enrichment calculation”). Functional annotation charts were generated using DAVID default stringency settings (“moderate”). Each downloaded chart was a spreadsheet containing a list of all known annotations that contained at least one gene from the input list of PE genes from the *t*-test and the probability that the input list of PE genes was enriched in genes of that annotation. Final annotation charts were then assembled by merging the UP- and DOWN-enriched annotations for each combination of PE dose and time. These charts were then reduced in complexity by discarding annotation terms that were not significantly enriched in the input PE gene list (*t*-test, Benjamini and Hochberg values >0.05) and by discarding all Gene Ontology (GO) terms in the DAVID output regardless of statistical significance. GO term annotations greatly increased the length of the annotation lists but have limited investigative value in the opinion of the authors. All microarray data have been deposited in the NIH/NCBI “GEO” database (http://www.ncbi.nlm.nih.gov/projects/geo; GEO accession number GSE13535).

## Results

### Six-group ANOVA, gene clustering, and pair-wise t-tests of treatment groups

**Fig. 1:**
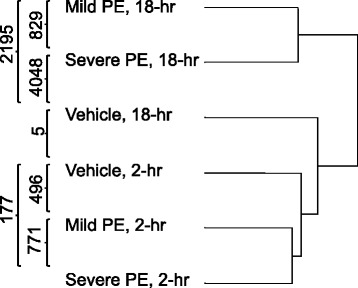
Hierarchical clustering of six treatment groups and results of pair-wise t-tests. Expression data from the six treatment groups were first compared by 2-way ANOVA using PE time as factor-1 and microsphere dose as factor-2. Relationships among the six treatments were determined using GeneSifters hierarchical clustering function applied to the 8075 Affymetrix probesets that passed the 2-way ANOVA. The separation of the six treatment groups on the dendrogram is based on relative Euclidean distance. Numerical data superimposed on the dendrogram are the results of pair-wise t-tests between the treatment groups

A comprehensive summary of all microarray data was first generated by comparing the six treatment groups (Mild PE, Severe PE and Vehicle each at 2-h and 18-h) in this study using a 2-way ANOVA, using the GeneSifter software suite (1.5-fold expression change minimum, Benjamini and Hochberg correction for false discovery, *p* < 0.05). The data is contained in Additional file 1 and shows that 8075 Affymetrix probesets passed the ANOVA for at least one of the tests (Factor 1, time; Factor 2, dose; interaction). The data from the ANOVA was then subjected to hierarchical clustering using GeneSifter to define the relationships between the six treatment groups. This output is summarized in the dendrogram shown in Fig. 1. The three 2-h groups were closely associated, as were the 18-h Mild PE and 18-h Severe PE groups while the 18-h vehicle clustered with the 2-h vehicle.

The six groups were also compared pair-wise (*t*-test, 1.5-fold expression change minimum, Benjamini and Hochberg correction, *p* < 0.05). These results are summarized on the left side of Fig. 1, and are available as spreadsheets in Additional file 2A-C and D-F (2-h groups and 18-h groups, respectively). For the Mild PE treatment groups there was a 4.43-fold increase in the number of significantly altered probesets between 2 and 18 h after PE (496 verses 2195). Similarly, for the Severe PE treatment groups there was 22.9-fold increase in the number of significantly altered probesets between 2 and 18 h after PE (177 verses 4048). Comparison of the Mild PE and Severe PE treatment groups at the same times revealed that the 18 h Severe PE group had 1.84-fold more altered probesets than the 18 h Mild PE group (4048 verses 2195), while the Severe PE group had fewer altered probesets at 2 h than the Mild PE group (177 verses 496). In a separate comparison of the two vehicle control groups (2 h and 18 h), only five probesets were significantly different, three of which were annotated in the GeneSifter database as “transcribed loci” only (data not shown). Taken together, these data indicated that although Severe PE produced a larger change in gene expression than Mild PE, Mild PE was sufficient to produce a robust change in lung gene expression.

### Separate analyses of 2-h and 18-h treatment groups

**Fig. 2:**
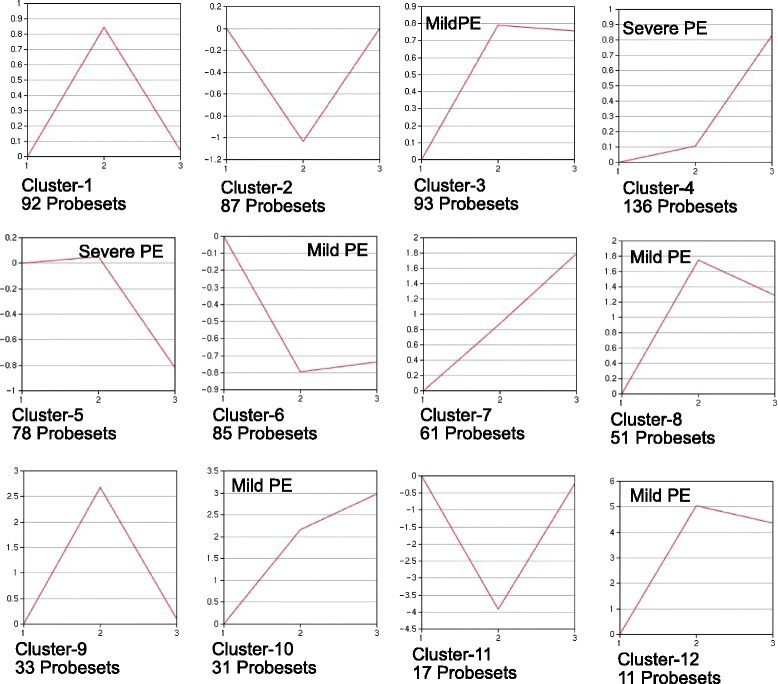
Clustering of 2-h treatment groups. Expression data from the three 2-h treatment groups (Vehicle, Mild PE, Severe PE; x-axis labels 1, 2, 3, respectively) were clustered using the PAM function of GeneSifter. A 12-cluster output was manually specified. Expression relative to Vehicle groups is provided on y-axes and is log2 transformed. The labels “Mild-PE” and “Severe-PE” are used to refer to patterns of expression that are primarily altered between the Vehicle and Mild PE groups or Mild PE and Severe PE groups, respectively

**Fig. 3:**
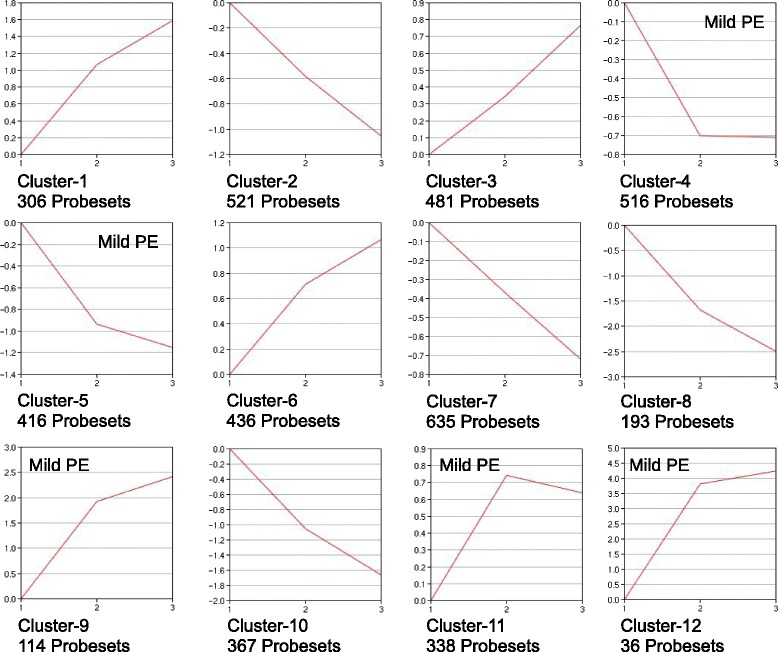
Clustering of 18-h treatment groups. Expression data from the three 18-h treatment groups (Vehicle, Mild PE, Severe PE; x-axis labels 1, 2, 3, respectively) were clustered as for Fig. 2

The responses of rats to mild PE and Severe PE were compared using separate 1-way ANOVAs of the 2-h and 18-h treatments with GeneSifter (expression difference threshold >1.5-fold, *t*-test <0.05, Benjamini and Hochberg). For the three 2-h treatment groups 775 probesets were altered in expression relative to the 2-h vehicle control, while 4360 probesets were altered in the 18-h groups relative to the 18-h vehicle control (1.5-fold expression difference threshold, *t*-test <0.05, Benjamini and Hochberg). The altered probesets in the 2-h and 18-h ANOVAs were then clustered into similar expression patterns with GeneSifter using the PAM function (Partitioning Around Medoids) with a user-designated output of 12 clusters for each ANOVA. These data are presented in Figs. 2 and 3 for 2-h and 18-h treatment groups, respectively.

The PAM-clustering of the 2-h ANOVA data is shown in Fig. 2, and clearly indicates three distinct patterns. 5 of 12 clusters had a “Mild PE-selective” pattern (clusters 3, 6, 8, 10 and 12). Probesets in these clusters had greater changes in expression between the vehicle and Mild PE samples than between the Mild PE and Severe PE samples. 2 of 12 clusters had a “Severe PE-selective” pattern (clusters 4 and 5). Probesets in these clusters had greater changes in expression between the Mild PE and Severe PE samples than between the vehicle and Mild PE samples. Curiously, 4 of 12 clusters had a Mild PE-specific pattern (clusters 1, 2, 9 and 11). Probesets in these clusters had peak increases or decreases in expression between the Vehicle and Mild PE treatments but minimal change in expression between the Mild PE ad Severe PE treatments. The magnitudes of the Mild PE expression changes in two of these clusters were within a single log2 increment of the vehicle and Severe PE groups (clusters 1 and 2) while the magnitudes of the Mild PE expression changes in the other two clusters (9 and 11) reached 4 log2 increments of the vehicle and Severe PE groups. These four clusters, and clusters 9 and 11 in particular, suggest that there were opposing signals for up- and down-regulation of gene expression in the Mild PE and Severe PE groups.

The PAM-clustering of the 18-h ANOVA data is shown in Fig. 3. Clustering of the 18-h treatment groups resulted in fewer clusters with obvious Mild PE-selective and Severe PE-selective gene expression than observed for the 2-h ANOVA. A plurality of clusters had “non-selective” patterns of gene expression (2, 3, 7, 8 and 10). Probesets in these clusters had nearly identical changes in expression between the vehicle and Mild PE samples and Mild PE and Severe PE samples. 4 of 12 clusters had a Mild PE-selective pattern (clusters 4, 5, 11 and 12) while two clusters (1 and 9) were intermediate between Mild PE-selective and non-selective. In contrast to the 2-h treatments, none of the clusters were indicative of Severe PE-selective expression.

### Enrichment of PE genes in functional annotations

Gene expression data from the pairwise t-tests of Mild PE and Severe PE treatment groups (Additional file 2A – F) were examined for significant enrichment of PE-altered probesets present in database annotations using DAVID web-based software (KEGG pathways, INTERPRO, SMART and SP_PIR database terms; GO terms excluded). Tables 1 and 2 summarize the functional annotations enriched in the Mild PE and Severe PE treatment groups, respectively.

**Table 1:** DAVID Functional Annotation Charts, 2-h and 18-h Mild PE verses Vehicle

Category	Term	^a^2-h Mild-PE vs. Vehicle					18-h Mild-PE vs. Vehicle				
		Ct	%	Dir	Fold	B&H	Ct	%	Dir	Fold	B&H
SP_PIR	acetylation						78	14.03	UP	1.51	0.009
***rno04920***	***Adipocytokine signaling pathway***	***6***	***2.93***	***UP***	***7.47***	***0.015***					
rno00970	Aminoacyl-tRNA biosynthesis						8	1.44	UP	5.56	0.017
***rno04210***	***Apoptosis***	***6***	***2.93***	***UP***	***5.89***	***0.028***					
rno00330	Arginine and proline metabolism						9	1.62	UP	4.72	0.015
rno05217	Basal cell carcinoma						10	1.11	D	4.28	0.016
IPR004827	Basic-leucine zipper (bZIP) transcription factor	7	3.41	UP	11.97	0.003					
SM00338	BRLZ	7	3.41	UP	10.46	0.001					
***rno04062***	***Chemokine signaling pathway***	***12***	***5.85***	***UP***	***5.85***	***0.000***	***20***	***3.60***	***UP***	***3.25***	***0.001***
SP_PIR	chemotaxis	9	4.39	UP	22.67	0.000	11	1.98	UP	10.30	0.000
rno04610	Complement and coagulation cascades						10	1.80	UP	3.97	0.019
SP_PIR	cytokine	11	5.37	UP	9.02	0.000	16	2.88	UP	4.88	0.000
***rno04060***	***Cytokine-cytokine receptor interaction***	***10***	***4.88***	***UP***	***4.24***	***0.008***	***23***	***4.14***	***UP***	***3.25***	***0.000***
***rno04623***	***Cytosolic DNA-sensing pathway***	***5***	***2.44***	***UP***	***9.07***	***0.024***					
***SP_PIR***	***disulfide bond***						***76***	***13.67***	***UP***	***1.43***	***0.044***
SP_PIR	dna-binding	20	9.76	UP	2.43	0.023					
rno00982	Drug metabolism						13	1.45	D	3.94	0.004
rno04512	ECM-receptor interaction						14	1.56	D	3.77	0.005
PIRSF001719	fos transforming protein	4	1.95	UP	59.84	0.001					
IPR000837	Fos transforming protein	4	1.95	UP	40.08	0.010					
rno00480	Glutathione metabolism						9	1.00	D	3.93	0.042
***rno04640***	***Hematopoietic cell lineage***	***6***	***2.93***	***UP***	***6.42***	***0.022***					
rno00340	Histidine metabolism						7	0.78	D	6.37	0.017
SP_PIR	inflammatory response	8	3.90	UP	16.01	0.000	11	1.98	UP	8.18	0.000
***rno04010***	***MAPK signaling pathway***	***10***	***4.88***	***UP***	***3.14***	***0.029***					
rno00980	Metabolism of xenobiotics by cytochrome P450						13	1.45	D	4.73	0.002
rno04621	NOD-like receptor signaling pathway	9	4.39	UP	12.11	0.000	10	1.80	UP	4.49	0.016
***SP_PIR***	***oxidoreductase***						***42***	***4.68***	***D***	***1.89***	***0.034***
***rno05020***	***Prion diseases***						***7***	***1.26***	***UP***	***5.56***	***0.028***
SP_PIR	ribosome biogenesis						7	1.26	UP	8.85	0.007
SM00199	SCY	8	3.90	UP	16.89	0.000	10	1.80	UP	8.76	0.000
***IPR000827***	***Small chemokine, C-C group, conserved site***	***4***	***1.95***	***UP***	***25.50***	***0.033***					
IPR001811	Small chemokine, interleukin-8-like	8	3.90	UP	19.35	0.000	10	1.80	UP	8.70	0.001
***PIRSF001950***	***small inducible chemokine, C/CC types***	***5***	***2.44***	***UP***	***24.93***	***0.001***					
***rno04620***	***Toll-like receptor signaling pathway***	***9***	***4.39***	***UP***	***8.34***	***0.000***					

Functional annotations significantly over-represented in the lists of up-regulated and down-regulated genes during 2-h and 18-h Low-PE. Annotations unique to Low-PE are highlighted in bold italics; annotations common to Low-PE and High-PE (Table 2) are in standard font. Key: “Ct.”, number genes from a GeneSifter pairwise *t*-test result (Additional file 3A-C) that were present in the functional annotation indicated; “%”, percent of genes contained within a list that were present in an annotation; “Dir, UP”, annotations that were identified by DAVID when up-regulated genes were used as the search query; “Dir, D”, annotations that were identified by DAVID when down-regulated genes were used as the search query. “Fold”, expression relative to vehicle group; B&H, value of Benjamini and Hochberg adjustment for false discovery following *t*-test. ^a^No 2-h DOWN annotations meet B&H < 0.05

**Table 2:** DAVID Functional Annotation Charts, 2-h and 18-h Severe PE verses Vehicle

Category	Term	^a^2-h Severe PE verses Vehicle					18-h Severe PE verses Vehicle				
		Ct	%	Dir	Fold	B&H	Ct	%	Dir	Fold	B&H
SP_PIR	acetylation						183	17.72	UP	1.86	0.000
***SP_PIR***	***activator***	***7***	***7.29***	***UP***	***5.58***	***0.025***					
***SP_PIR***	***acute phase***						***7***	***0.68***	***UP***	***4.89***	***0.047***
***rno00520***	***Amino sugar and nucleotide*** ***sugar metabolism***						***12***	***1.16***	***UP***	***4.37***	***0.002***
rno00970	Aminoacyl-tRNA biosynthesis						12	1.16	UP	4.70	0.002
SP_PIR	Aminoacyl-tRNA synthetase						10	0.97	UP	5.11	0.003
rno00330	Arginine and proline metabolism						11	1.06	UP	3.25	0.046
***rno05412***	***Arrhythmogenic right ventricular*** ***cardiomyopathy (ARVC)***						***15***	***0.96***	***D***	***2.56***	***0.040***
rno05217	Basal cell carcinoma						12	0.77	D	2.97	0.036
IPR011700	Basic leucine zipper	4	***4.17***	***UP***	***41.80***	***0.004***					
IPR004827	Basic-leucine zipper (bZIP) transcription factor	8	8.33	UP	28.54	0.000					
SM00338	BRLZ	8	8.33	UP	20.66	0.000					
IPR011616	bZIP transcription factor, bZIP-1	4	4.17	UP	25.44	0.015					
***SP_PIR***	***Chaperone***						***18***	***1.74***	***UP***	***2.78***	***0.006***
SP_PIR	chemotaxis	4	4.17	UP	20.78	0.025	9	0.87	UP	4.43	0.016
***SP_PIR***	***Cholesterol biosynthesis***						***9***	***0.87***	***UP***	***7.47***	***0.000***
rno04610	Complement and coagulation cascades						17	1.65	UP	3.80	0.000
SP_PIR	cytokine	9	9.38	UP	15.21	0.000					
SP_PIR	DNA binding	6	6.25	UP	7.38	0.028					
SP_PIR	dna-binding	15	15.63	UP	3.75	0.002					
***UP_SEQ***	***DNA-binding region: Basic motif***	***10***	***10.42***	***UP***	***15.44***	***0.000***					
***UP_SEQ***	***domain: Leucine-zipper***	***9***	***9.38***	***UP***	***19.96***	***0.000***					
rno00982	Drug metabolism						20	1.28	D	3.51	0.000
rno04512	ECM-receptor interaction						17	1.09	D	2.65	0.020
***SM00180***	***EGF_Lam***						***9***	***0.58***	***D***	***5.44***	***0.020***
***IPR002049***	***EGF-like, laminin***						***9***	***0.58***	***D***	***5.98***	***0.026***
***SP_PIR***	***endoplasmic reticulum***						***63***	***6.10***	***UP***	***2.04***	***0.000***
PIRSF001719	fos transforming protein	4	4.17	UP	109.2	0.000					
IPR000837	Fos transforming protein	4	4.17	UP	83.59	0.001					
rno00480	Glutathione metabolism						12	0.77	D	3.03	0.041
rno00340	Histidine metabolism						9	0.58	D	4.73	0.019
SP_PIR	inflammatory response	4	4.17	UP	16.51	0.027	13	1.26	UP	5.08	0.000
***SP_PIR***	***Initiation factor***						***12***	***1.16***	***UP***	***4.55***	***0.002***
***SP_PIR***	***Isomerase***						***17***	***1.65***	***UP***	***3.14***	***0.003***
***SP_PIR***	***lipid synthesis***						***19***	***1.84***	***UP***	***3.82***	***0.000***
rno00980	Metabolism of xenobiotics by cyt. P450						18	1.15	D	3.79	0.000
***UP_SEQ***	***mutagenesis site***						***59***	***3.77***	***D***	***1.75***	***0.042***
rno04621	NOD-like receptor signaling pathway	5	5.21	UP	12.52	0.034					
***SP_PIR***	***nucleotide-binding***						***99***	***9.58***	***UP***	***1.36***	***0.024***
SP_PIR	nucleus	22	22.92	UP	2.01	0.026					
***SP_PIR***	***phosphoprotein***						***##***	***27.49***	***UP***	***1.24***	***0.000***
***IPR011993***	***Pleckstrin homology-type***						***32***	***2.05***	***D***	***2.27***	***0.027***
***SP_PIR***	***protein biosynthesis***						***22***	***2.13***	***UP***	***2.04***	***0.049***
***SP_PIR***	***protein transport***						***34***	***3.29***	***UP***	***1.87***	***0.016***
***SP_PIR***	***Redox-active center***						***10***	***0.97***	***UP***	***4.74***	***0.005***
SP_PIR	ribosome biogenesis						8	0.77	UP	5.31	0.014
***SP_PIR***	***rna-binding***						***33***	***3.19***	***UP***	***2.16***	***0.002***
***SM00360***	***RRM***						***21***	***2.03***	***UP***	***2.69***	***0.015***
SM00199	SCY	5	5.21	UP	18.26	0.003					
IPR001811	Small chemokine, interleukin-8-like	5	5.21	UP	25.22	0.002					
***SP_PIR***	***Steroid biosynthesis***						***13***	***1.26***	***UP***	***5.08***	***0.000***
***rno00100***	***Steroid biosynthesis***						***9***	***0.87***	***UP***	***8.29***	***0.001***
***SP_PIR***	***sterol biosynthesis***						***11***	***1.06***	***UP***	***7.31***	***0.000***
***SP_PIR***	***Transcription***	***14***	***14.58***	***UP***	***3.23***	***0.009***					
***SP_PIR***	***transcription regulation***	***14***	***14.58***	***UP***	***3.48***	***0.006***					
***SP_PIR***	***translocation***						***11***	***1.06***	***UP***	***3.75***	***0.014***
***rno00350***	***Tyrosine metabolism***						***10***	***0.64***	***D***	***3.71***	***0.032***

Functional annotations significantly over-represented in the lists of up-regulated and down- regulated genes during 2-h and 18-h High-PE. Annotations unique to High-PE are highlighted in bold italics; annotations common to High-PE and Low-PE (Table 1) are in standard font. All other keys are the same as in Table 1. ^a^No 2-h DOWN annotations meet B&H < 0.05

Functional annotations enriched in the Mild PE treatment groups relative to time-matched vehicle controls are shown in Table 1. Annotations unique to Mild PE treatment groups are highlighted with bold font and italics while annotations common to Low- and Severe PE are in standard font. Blank cells in the data indicate that an annotation was not significantly over-represented at a particular time and treatment combination. At the 2-h time, the majority of annotations (10 of 21 total annotations) were unique to Mild PE, while a minority (11 of 21) were shared with the Severe PE 2-h group (Table 2). Conversely, at 18-h only 5 of 22 total annotations were unique to Mild PE (23 %) while 17 of 22 (77 %) were shared with the Severe PE group (Table 2). These data support a distinct mechanistic difference between Mild and Severe PE. If Mild PE were merely a less robust manifestation of Severe PE, all annotations present during Mild PE would be expected to be present during Severe PE.

Functional annotations enriched in the Severe PE treatment groups relative to time-matched vehicle controls are shown in Table 2. Annotations unique to Severe PE treatment groups are highlighted with bold font and italics while annotations common to Low PE and Severe PE are in standard font. It is noteworthy that the data in Table 2 showed a distinct separation of annotations between the two times. Of the 57 total annotations listed in the table only one, “SP-PIR chemotaxis”, was present at both times. The remaining 56 annotations were present at only one of the time points. This pattern was not seen with the Low PE data in Table 1 in which a greater proportion of the total annotations were present at both times. Together, these data suggest a rapid “progression” of the Severe PE condition at the transcriptional level compared to a more gradual transcriptional “progression” for Mild PE.

### Annotations unique to mild PE

Further examination of the Mild PE expression data in Table 2 revealed that 13 of 35 total annotations were unique to Mild PE. Several of these unique annotations involved pro-inflammatory KEGG pathways or protein families. The KEGG pathways were: rno04920 “adipocytokine signaling”, rno04062 “chemokine signaling”, rno04060 “cytokine-cytokine receptor”, and rno04620 “Toll-like receptor signaling”. Rno04010 “MAPK signaling pathway” was also present, but this pathway intersects with diverse cellular processes beside inflammation. The Interpro annotation IPR000827 “small cytokine C-C” and Protein Information Resource annotation PIRSF001950 “small inducible chemokine” were also present. The presence of gene annotations unique to the 2-h Mild PE treatment, which shares little physiological similarity with Severe PE, continue to support a conclusion that Mild PE and Severe PE may be mechanistically dissimilar at the transcriptional level.

### Are mild PE and severe PE related?

The possible similarities between the 18-h Mild PE and 2-h Severe PE found in Tables 1 and 2 prompted a direct comparison between these two treatment groups. These data are presented in Table 3. However, only 6 of 36 annotations present in either of the two treatments were shared by both: SP-PIR chemotaxis, SP_PIR cytokine, SP_PIR “inflammatory response”, rno04621 “NOD-like receptor signaling pathway”, SM00199 “SCY” and IPR001811 “small chemokine interleukin-8-like”. The remaining 31 annotations which were enriched in either the 2-h Severe PE or 18-h Mild PE groups were confined to one or the other group. These data refute a similarity between early Severe PE and late Mild PE.

**Table 3:** DAVID Functional Annotation Charts, 2-h Severe PE and 18-h Mild PE verses Vehicle

		^a^2-h Severe-PE verses Vehicle					18-h Mild-PE verses Vehicle				
Category	Term	Ct	%	Dir	Fold	B&H	Ct	%	Dir	Fold	B&H
SP_PIR	acetylation						78	14.03	UP	1.51	0.009
SP_PIR	activator	7	7.29	UP	5.58	0.025					
rno00970	Aminoacyl-tRNA biosynthesis						8	1.44	UP	5.56	0.017
rno00330	Arginine and proline metabolism						9	1.62	UP	4.72	0.015
rno05217	Basal cell carcinoma						10	1.11	D	4.28	0.016
IPR011700	Basic leucine zipper	4	4.17	UP	41.80	0.004					
IPR004827	Basic-leucine zipper (bZIP) transcription factor	8	8.33	UP	28.54	0.000					
SM00338	BRLZ	8	8.33	UP	20.66	0.000					
IPR011616	bZIP transcription factor, bZIP-1	4	4.17	UP	25.44	0.015					
rno04062	Chemokine signaling pathway						20	3.60	UP	3.25	0.001
***SP_PIR***	***chemotaxis***	***4***	***4.17***	***UP***	***20.78***	***0.025***	***11***	***1.98***	***UP***	***10.30***	***0.000***
rno04610	Complement and coagulation cascades						10	1.80	UP	3.97	0.019
***SP_PIR***	***cytokine***	***9***	***9.38***	***UP***	***15.21***	***0.000***	***16***	***2.88***	***UP***	***4.88***	***0.000***
rno04060	Cytokine-cytokine receptor interaction						23	4.14	UP	3.25	0.000
SP_PIR	disulfide bond						76	13.67	UP	1.43	0.044
SP_PIR	DNA binding	6	6.25	UP	7.38	0.028					
SP_PIR	dna-binding	15	15.63	UP	3.75	0.002					
UP_SEQ	DNA-binding region: Basic motif	10	10.42	UP	15.44	0.000					
UP_SEQ	domain: Leucine-zipper	9	9.38	UP	19.96	0.000					
rno00982	Drug metabolism						13	1.45	D	3.94	0.004
rno04512	ECM-receptor interaction						14	1.56	D	3.77	0.005
PIRSF001719	fos transforming protein	4	4.17	UP	109.20	0.000					
IPR000837	Fos transforming protein	4	4.17	UP	83.59	0.001					
rno00480	Glutathione metabolism						9	1.00	D	3.93	0.042
rno00340	Histidine metabolism						7	0.78	D	6.37	0.017
***SP_PIR***	***inflammatory response***	***4***	***4.17***	***UP***	***16.51***	***0.027***	***11***	***1.98***	***UP***	***8.18***	***0.000***
rno00980	Metabolism of xenobiotics by cytochrome P450						13	1.45	D	4.73	0.002
***rno04621***	***NOD-like receptor signaling pathway***	***5***	***5.21***	***UP***	***12.52***	***0.034***	***10***	***1.80***	***UP***	***4.49***	***0.016***
SP_PIR	nucleus	22	22.92	UP	2.01	0.026					
SP_PIR	oxidoreductase						42	4.68	D	1.89	0.034
rno05020	Prion diseases						7	1.26	UP	5.56	0.028
SP_PIR	ribosome biogenesis						7	1.26	UP	8.85	0.007
***SM00199***	***SCY***	***5***	***5.21***	***UP***	***18.26***	***0.003***	***10***	***1.80***	***UP***	***8.76***	***0.000***
***IPR001811***	***Small chemokine, interleukin-8-like***	***5***	***5.21***	***UP***	***25.22***	***0.002***	***10***	***1.80***	***UP***	***8.70***	***0.001***
SP_PIR	Transcription	14	14.58	UP	3.23	0.009					
SP_PIR	transcription regulation	14	14.58	UP	3.48	0.006					

Functional annotations significantly over-represented in the lists of up-regulated and down-regulated genes during 2-h Severe PE and 18-h Mild PE. Annotations unique to both treatments are highlighted in bold Italics; unique annotations are in standard font. Keys are the same as in Table 1. ^a^No 2-h DOWN Annotations meet B&H < 0.05

### Steroid synthesis during 18-h severe PE

Three annotations were detected in the 18-h Severe PE gene lists which were associated with steroid and/or sterol biosynthesis. A total of 16 unique genes were contained within these annotations. These data are presented in Table 4. Most of these genes were contained on the KEGG pathway rno00100 (R. norvegicus Steroid Biosynthesis; http://www.kegg.jp/kegg-bin/show_pathway?rno00100). This pathway terminates with several branches but all of the genes induced by Severe PE were located on the branch terminating with cholesterol.

**Table 4:** Steroid/Sterol Biosynthesis Genes

Symbol	Fold	Dir.	Gene Name
CH25H	6.84	UP	Cholesterol 25-hydroxylase
DHCR24	2.33	UP	24-dehydrocholesterol reductase
DHCR7	2.15	UP	7-dehydrocholesterol reductase
FDFT1	1.56^a^	UP	Farnesyl diphosphate farnesyl transferase 1
FDPS	1.89	UP	Farnesyl diphosphate synthase (farnesyl pyrophosphate synthetase, dimethylallyltranstransferase, geranyltranstransferase)
HMGCR	2.11^a^	UP	3-hydroxy-3-methylglutaryl-Coenzyme A reductase
HMGCS1	3.74	UP	3-hydroxy-3-methylglutaryl-Coenzyme A synthase 1 (soluble)
HSD17B12	3.03	UP	Hydroxysteroid (17-beta) dehydrogenase 12
HSD17B7	4.33	UP	Hydroxysteroid (17-beta) dehydrogenase 7
IDI1	8.61	UP	Isopentenyl-diphosphate delta isomerase 1
MVD	3.34	UP	Mevalonate (diphospho) decarboxylase
NSDHL	1.84	UP	NAD(P) dependent steroid dehydrogenase-like
SC4MOL	1.9	UP	Sterol-C4-methyl oxidase-like
SC5DL	2.33^a^	UP	Sterol-C5-desaturase (ERG3 delta-5-desaturase homolog, S. cerevisiae)
SOAT1	2.59^a^	UP	Sterol O-acyltransferase 1
SQLE	3.26	UP	Squalene epoxidase

Steroid and sterol biosynthesis genes altered in the 18-h Severe PE treatment group. The three steroid/sterol annotations from Table 2 contained a total of 16 unique genes. All of the original DAVID downloads of PE-regulated annotations shown in Tables 1 and 2 and included lists of the genes within an annotation that were also altered by PE. These gene lists were very large and were not included in Tables 1, 2 and 3 to save space. The three annotations “SP_PIR Steroid biosynthesis”, “rno00100 Steroid biosynthesis” and “SP_PIR Sterol biosynthesis” contained 13, 9 and 11 genes, respectively. The 16 genes shown above represent the total unique genes. “Dir”, direction of fold-change. ^a^Average fold-change of 2 or more probesets for a single gene

HMGCR, HMGCS1 and IDI1 function upstream of rno00100 and were found on the KEGG pathway rno00900 (R. norvegicus Terpenoid Backbone Biosynthesis; http://www.kegg.jp/kegg-bin/show_pathway?rno00900). This pathway begins with acetyl-CoA, proceeds through 3-hydroxy-3-methyglutaryl-CoA (HMG-CoA), mevalonate, isopentenyl-diphosphate and farnesyl-diphosphate, which is the metabolic intermediate that feeds KEGG Pathway rno00100.

## Discussion

Microsphere-induced PE caused profound changes in gene expression in rat lungs even when RVSP was lower than typically considered clinically relevant. These data contrast sharply with previous results on the effects of this same PE model on transcriptional changes in hearts [23]. In this latter study, Zagorski et al. demonstrated an almost obligatory requirement for PH to cause altered gene expression in RV tissues [23]. In particular, 2-h Mild PE resulted in no statistically significant transcriptional changes in RVs and few changes after 18-h. The effect of Mild PE on gene expression in lung tissue reported here was much more dramatic. 2-h of Mild PE with minimal PH was sufficient to cause numerous changes in gene expression and statistically significant alteration in at least 21 expression pathways or other gene group annotations. Examination of the annotations enriched in the 2-h Mild PE “UP” gene expression data revealed several pro-inflammatory annotation terms. The abundance of pro-inflammatory annotations enriched in 2-h Mild PE samples indicates that lungs exposed to even mild PE with minimal PH rapidly enter into a pro-inflammatory state. This data supports a conclusion that even mild PE has the potential to initiate damage to lung tissues. This is in stark contrast to the total lack of right ventricular inflammation seen in the microsphere model of Mild PE [19–25].

Mild and Severe PE are hemodynamically distinct at 2-h post-PE, with only the latter having PH. This brings into question the stimuli imparted on lungs with 2-h Mild PE which are responsible for the large changes in gene expression. The most likely explanation is ischemia, which is often considered synonymous with hypoxia. However, ischemia resulting from occlusion of the pulmonary vasculature is unique, since the pulmonary artery circulates oxygen-poor and nutrient-depleted blood derived from the venous circulation. In essence, even normal circulation through the pulmonary artery is hypoxic, and occluded flow through the pulmonary artery has no effect on oxygenation of lung tissue, which is dependent on the separate bronchial circulation. Furthermore, lung tissue is continuously exposed to atmospheric oxygen via the airways. It seems unlikely that hypoxia accounts for the early lung transcriptional response to Mild PE. Supporting evidence for this conclusion is contained in this study. Expression of the hypoxia marker genes Hif1 (hypoxia-inducible factor-1) and Hyou1 (hypoxia up-regulated 1) were up-regulated in the 18-h Severe PE treatment group by modest 1.6851-fold and 3.3772-fold levels, respectively (Additional file 2E; lines 6823 and 4267) but neither was up-regulated by the Mild PE treatment. These results discount a role for lung hypoxia following PE, at least at the Mild PE dose of microspheres.

It seems reasonable that some hemostatic disruption related to ischemia, but not based on transfer of oxygen and/or nutrients to lung tissue, was responsible for the effects on gene expression seen in this study. An intriguing possibility is “stop-of-flow”, a phenomenon introduced by Fisher and co-workers [29–34]. Vascular endothelium are adapted to conditions of flow in vivo, with cell membranes and cytoskeletons aligning along the axis of blood flow. This can also be mimicked in vitro by applying flow over cells which were initially cultured in the absence of flow [33]. Endothelial cells grown in the absence of flow show a random organization of membranes and cytoskeletal structures, but when a laminar flow is applied to these cultures the cells adopt a flow-axial organization similar to that seen in vivo. Importantly, when flow is discontinued on cultures of flow-adapted cells, several signaling pathways are activated, including a signaling cascade mediated by NADPH oxidase 2-dependent ROS production (reactive oxygen species; 32). This also occurs in vivo in isolated perfused lungs subjected to ischemia. Pulmonary endothelial membrane depolarization, H2O2 production and increased intracellular Ca2+ have been observed within 10–15 s after the onset of non-hypoxic ischemia [34]. This rapid response can easily accommodate the increased gene expression seen in rats with 2-h Mild PE. Several comprehensive reviews have been published on the proposed general applicability of the “stop-of-flow” mechanism to explain the pathophysiology of tissue ischemia, including the ischemia associated with PE [35–37].

Finally, 18-h Severe PE resulted in the over-expression of genes present in several gene annotations related to steroid, lipid, and/or cholesterol biosynthesis. These results are consistent with a recent report that bile acids accumulate in lung tissues suffering from pulmonary artery hypertension; bile acids are downstream of cholesterol biosynthesis [38]. They are also consistent with numerous studies that have demonstrated the efficacy HMG-CoA reductase inhibitors (statins) in reducing the severity of pulmonary hypertension in both chronic hypoxia and monocrotaline animal models [39–44], although contradictory results have also been observed [45]. Several mechanisms, un-linked to the known effect of statin drugs on reducing serum cholesterol, have been proposed to explain the efficacy of statins for PH but a clear explanation is premature.

Clinical relevance includes the potential hypothesis that PE without PH may produce inflammatory changes in the lung, leading to lung-initiated, systemic inflammation, and increased risk of ongoing hypercoagulability and clot recurrence [13, 14]. Humans with PE have 4–7 fold increases in circulating biomarkers of inflammation (tumor necrosis factor, C reactive protein, interleukin 6 and myeloperoxidase) that return to near normal levels after three months of treatment [46, 47]. Approximately 1/3 of patients with PE have sustained unresolved perfusion defects after PE diagnosis [48]. A growing body of literature has found that treatment with statins reduce circulating concentrations of IL-6, CRP and monocyte chemoattractant protein 1, and may reduce VTE recurrence [49]. Our data suggest persistent pulmonary vascular occlusion, even with minimal or absent PH may cause lung inflammation with PE. These data may imply that early re-canalization should decrease this inflammatory response. This is a prime goal of “Pulmonary Embolism Response Teams” being organized at many sites around the US with the goal of rapid recanalization of pulmonary arteries in patients with severe PE [50].

## Conclusion

This is the first report to show that mild pulmonary embolism produces profound alteration in gene transcription in lungs, primarily in terms of increased expression of genes encoding inflammatory chemokines and cytokines and cholesterol synthesis. These data show that unresolved pulmonary vascular occlusion produces ongoing lung inflammation even in the absence of elevated pulmonary arterial pressures. Translational implications include the adverse effects of ongoing inflammation from unresolved pulmonary vascular occlusions, and conversely, possible benefit of treating PE to an endpoint of complete clot resolution.

## Additional files

Additional file 1: Rat lung 6-group 2-way ANOVA. (XLS 3757 kb) Additional file 2: Pairwise t-test of PE treatments. (XLS 2775 kb) Additional file 3: ANOVAs of 2-hour and 18-hour PE treatments. (XLS 1660 kb)
